# Magnesium in Neurocritical Care: Clinical Relevance, Status Assessment, and Practical Implications for Outcomes—A Narrative Review

**DOI:** 10.3390/nu18091359

**Published:** 2026-04-25

**Authors:** Stefano Marelli, Lorenzo Querci, Arturo Chieregato

**Affiliations:** Neurocritical Care Unit, ASST Grande Ospedale Metropolitano Niguarda, 20162 Milan, Italy; lorenzo.querci@ospedaleniguarda.it (L.Q.); arturo.chieregato@ospedaleniguarda.it (A.C.)

**Keywords:** magnesium sulfate, ionized magnesium, neurocritical care, traumatic brain injury, stroke, subarachnoid hemorrhage, magnesium homeostasis, dietary magnesium, neurologic outcomes

## Abstract

Background: Magnesium regulates neuronal excitability, NMDA receptor activity, and cerebrovascular tone. Dysmagnesemia is common in patients with acute brain injury (>65%), yet large randomized trials of magnesium neuroprotection have been neutral despite strong physiological rationale and consistent observational associations with outcomes. A key limitation may be diagnostic misclassification: the total serum magnesium poorly reflects the biologically active ionized fraction and may misclassify magnesium status in 20–85% of ICU patients during critical illness. Purpose: This narrative review synthesizes current evidence on magnesium physiology, measurement limitations, and clinical implications in neurocritical care. Overview: We discuss the mechanisms of magnesium depletion, outline the conceptual “two-hit” model (chronic deficiency plus acute ICU losses), and highlight the potential value of ionized magnesium for improved patient evaluation. Emerging syndrome-specific data suggest that magnesium disturbances are associated with prognostic signals. Improved phenotyping may help explain prior trial neutrality and support stratified approaches to magnesium monitoring and repletion. Future studies should evaluate magnesium-guided strategies and phenotype-driven trials to clarify the therapeutic role of magnesium in neurocritical care.

## 1. Introduction

Magnesium is a cation essential for membrane stability, neuromuscular transmission, vascular tone, mitochondrial function, and immunometabolism [1]. In neurocritical care, dysmagnesemia is common in acute brain injuries because traumatic brain injury (TBI), aneurysmal subarachnoid hemorrhage (aSAH), stroke, and intensive care interventions disrupt magnesium homeostasis [2,3,4]. This may contribute to extracranial and intracranial complications such as arrhythmias, vasospasm, and secondary brain injury [5,6]. Seven of ten large RCTs testing magnesium supplementation for neuroprotection in stroke, aSAH, and TBI have been neutral, despite strong preclinical rationale and observational links between low magnesium and poor outcomes. A key uncertainty is the measurement: the total serum magnesium (tMg) misclassifies biologically active ionized magnesium (iMg) in 20–60% of Intensive Care Unit (ICU) cases [7,8,9]. This misclassification is due to shifts in critical illness, leading to undertreatment or overtreatment without verified deficits [3,10,11]. This narrative review synthesizes magnesium’s roles in acute brain injury, clarifies assessment pitfalls, and evaluates outcome links across neurocritical syndromes to propose stratified, precision-guided management.

## 2. Materials and Methods

### 2.1. Search Strategy

A structured literature search was conducted in PubMed and Embase covering the period from 1 January 1984 to 31 December 2025. The search strategy combined MeSH terms and free-text terms related to magnesium and neurocritical conditions. The full search string, including terms for magnesium exposure, was reported in Appendix A. In addition, reference lists of relevant reviews and key articles were manually screened to identify further eligible studies.

### 2.2. Study Selection and Flow

**Figure A1:**
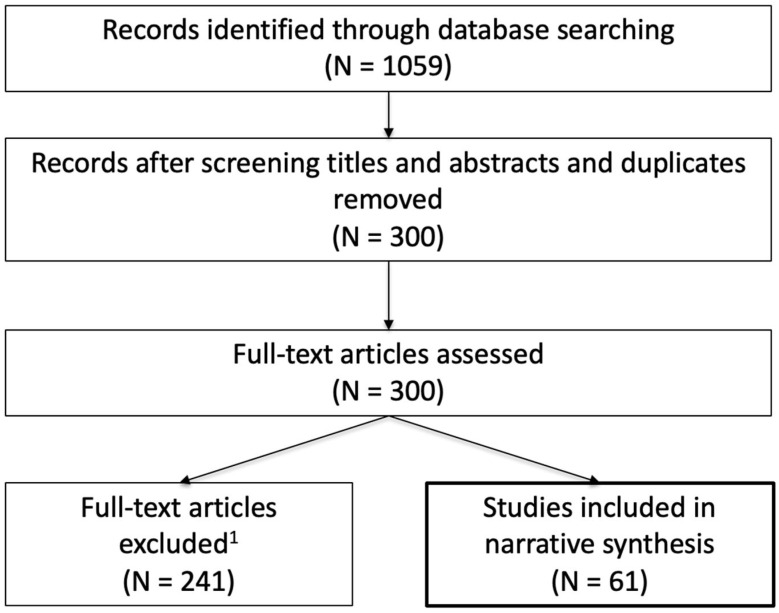
PRISMA-style flow diagram of study selection. A total of 1059 records were identified through systematic database searching of PubMed and Embase (1 January 1984–31 December 2025), supplemented by manual screening of reference lists. After removal of duplicates and title/abstract screening for relevance to magnesium physiology, measurement, and clinical outcomes in neurocritical or related intensive care populations, 300 articles were retained for full-text assessment. Of these, 241 articles were excluded based on predefined criteria, including the absence of clinically meaningful outcomes, non-neurocritical populations, redundant data from overlapping cohorts, or purely descriptive and mechanistic studies without direct clinical applicability. A total of 61 studies were included in the final narrative synthesis. ^1^ Full-text exclusions were primarily due to a lack of relevant clinical outcomes, non-neurocritical populations, redundant data from overlapping cohorts, or purely descriptive/mechanistic studies without direct clinical applicability. When multiple publications addressed overlapping datasets or concepts, priority was given to the most comprehensive or recent synthesis to avoid redundancy.

Study selection was performed in two stages by two clinicians. In the first stage, titles and abstracts were screened for relevance to magnesium physiology, measurement, and clinical outcomes in neurocritical or closely related intensive care populations. Articles clearly unrelated to neurocritical care, non-human studies without translational relevance, and studies lacking outcome data were excluded. In the second stage, full texts of potentially relevant articles were assessed. Studies were selected based on the relevance to neurocritical populations or critical illness, the availability of clinically meaningful outcomes, and contribution to methodological understanding of magnesium assessment. Full-text exclusions were primarily due to a lack of relevant clinical outcomes, non-neurocritical populations, redundant data from overlapping cohorts, or purely descriptive/mechanistic studies without direct clinical applicability. When multiple publications addressed overlapping datasets or concepts, priority was given to the most comprehensive or recent synthesis to avoid redundancy. Overall, 1059 records were identified through database searching. After removing duplicates and screening titles and abstracts, 300 articles were retained for full-text assessment. Of these, 241 articles were excluded after full-text evaluation based on predefined relevance and methodological criteria, leaving 61 studies included in the final narrative synthesis. A PRISMA-style flow diagram summarizing the selection process is provided (Figure A1, Appendix A).

### 2.3. Quality Appraisal and Synthesis Approach

Given the narrative nature of this review, a formal risk-of-bias assessment was not performed. However, study quality was implicitly considered during selection and synthesis, with prioritization of randomized controlled trials, large prospective or observational ICU datasets, and methodologically robust studies on magnesium measurement. Smaller mechanistic or proof-of-concept studies were included when they provided unique insights into physiology. The structure and interpretation of the review were guided by SANRA principles, with an emphasis on transparency in methodology, a balanced interpretation of evidence, and the integration of physiological and clinical perspectives.

## 3. Main Body

### 3.1. Relevant Magnesium Physiology in Acute Brain Injury

**Figure 1:**
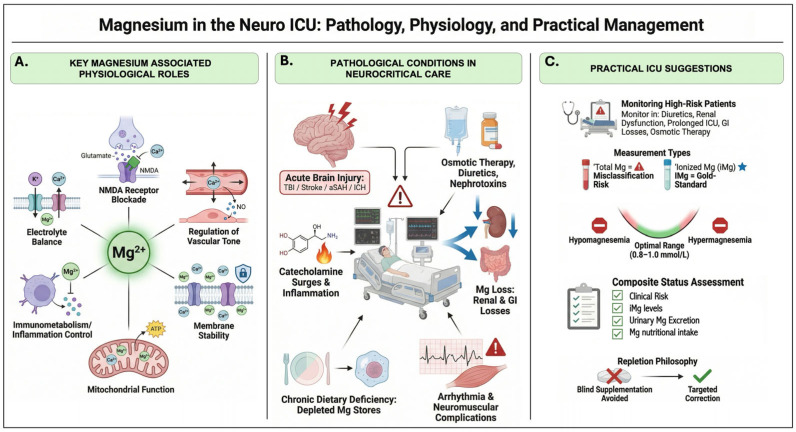
Magnesium in the neuro-ICU: key physiological roles, pathological conditions, and practical management considerations. (**A**) Core magnesium-associated physiological functions relevant to the central nervous system, including NMDA receptor blockade [12,13,14,15,16], regulation of vascular tone and cerebrovascular reactivity [17,18,19], electrolyte balance, membrane stability, mitochondrial function, and immunometabolic regulation. (**B**) Pathological conditions predisposing to magnesium dysregulation in neurocritical care, including acute brain injury (traumatic brain injury, stroke, aneurysmal subarachnoid hemorrhage, and intracerebral hemorrhage) [2,3,4,20,21,22,23,24,25,26,27,28,29,30,31,32,33,34,35,36,37,38,39,40,41,42,43,44,45,46,47,48], catecholamine surges and systemic inflammation, osmotic therapy, diuretics, and nephrotoxin exposure, renal and gastrointestinal losses, chronic dietary deficiency or depleted magnesium stores, and resultant arrhythmic and neuromuscular complications. (**C**) Practical ICU management suggestions, encompassing identification of high-risk patients, selection of measurement modality, with ionized magnesium as a potentially more biologically relevant marker than total magnesium [7,8,9,10,49,50], composite status assessment integrating clinical risk, ionized magnesium levels, urinary magnesium excretion, and nutritional intake, and a repletion philosophy favoring individualized, status-informed correction over empirical or uniform supplementation. iMg = ionized magnesium; aSAH = aneurysmal subarachnoid hemorrhage; ICH = intracerebral hemorrhage; TBI = traumatic brain injury; GI = gastrointestinal; ICU = intensive care unit.

Magnesium participates in a broad spectrum of physiological functions; including mitochondrial energy metabolism, membrane stability, electrolyte balance, and immunometabolic regulation, as illustrated in Figure 1A. Its most consequential roles in the context of acute brain injury, however, reside at the neuronal and cerebrovascular level: neuronal excitability and NMDA receptor gating; and vascular tone, microcirculation, and vasospasm biology.

#### 3.1.1. Neuronal Excitability and NMDA Receptor Gating

Magnesium exerts a voltage-dependent block on NMDA receptor channels, regulating Ca^2+^ influx and preventing excitotoxic injury cascades in central neurons. At resting membrane potentials, extracellular magnesium binds within the channel pore, blocking Ca^2+^ entry until depolarization relieves this inhibition in the presence of glutamate [12,13,14].

Building on this mechanism, ischemia or energy failure sustains membrane depolarization, thereby unleashing excessive NMDA-mediated Ca^2+^ influx. This triggers mitochondrial dysfunction, reactive oxygen species, and apoptosis. TBI models exacerbate this by mechanically weakening the block, amplifying ionic currents and intracellular Ca^2+^ overload, thereby worsening neuronal damage. Thus, magnesium homeostasis is pivotal, as its disturbance unmasks excitotoxicity, a central feature of acute brain injury progression [15,16].

#### 3.1.2. Vascular Tone, Microcirculation, and Vasospasm Biology

Magnesium acts as a physiological calcium antagonist in vascular smooth muscle, inhibiting Ca^2+^ influx via voltage-gated channels, activating K^+^ channels, and enhancing endothelial Nitric Oxide production to promote vasodilation and counteract vasospasm, particularly relevant in aSAH [17,18]. In cerebral arterioles, elevated extracellular magnesium dose-dependently dilates endothelium-impaired vessels and reverses constriction by spasmogens, such as endothelin-1, preserving microcirculatory flow and mitigating delayed cerebral ischemia [19].

### 3.2. A “Two-Hit” Model: From Chronic Low Intake to Acute ICU Magnesium Debt

Magnesium deficiency in neurocritical care frequently arises via a “two-hit” model, where “hit 1” comprises chronic suboptimal dietary intake and/or comorbidities (e.g., diabetes, alcoholism) that silently erode intracellular magnesium reserves despite normal serum levels, affecting up to 10–20% of the general population [11]. “Hit 2” delivers acute exacerbation from neurocritical stressors (catecholamine surges, inflammation, catecholamine-resistant shock) and ICU interventions (e.g., loop diuretics, proton pump inhibitors, nasogastric suction, sepsis), yielding >65% hypomagnesemia prevalence.

### 3.3. Assessing Magnesium Status: What We Measure vs. What We Need

In neurocritical care, total serum magnesium frequently misclassifies biologically active magnesium status. This discrepancy arises because the serum total magnesium (tMg) encompasses three biochemically distinct fraction: ionized (free, ~55–60%), protein-bound (~25–30%), and complexed (~15%; of these, only the ionized fraction is biologically active). Prevalent conditions in critical illness, such as hypoalbuminemia, acid–base shifts, and catecholamine surges, can reduce the ionized fraction while leaving tMg within the apparent normal range (Figure 1B) [11]. Analytical factors such as hemolysis, anticoagulants, and pH fluctuations further compound misclassification, and may contribute, among other factors, to suboptimal repletion strategies when assessment relies solely on tMg values. In contrast, ionized magnesium (iMg) measured via ion-selective electrodes directly captures the unbound, biologically active fraction and shows stronger associations with clinically relevant outcomes, including arrhythmias, mortality, and the ICU length of stay [1,10].

Complementary urinary indices, particularly the Fractional Excretion of Magnesium (FEMg > 2–4%), help differentiate true depletion from redistribution phenomena [51,52]. Variable reference limits for tMg (0.65–0.75 mmol/L) and iMg (0.45–0.55 mmol/L), plus the absence of harmonization, undermine research comparability and homogeneous clinical practice [11]. Advanced tools like stable isotopes (^25^Mg/^26^Mg) for absorption kinetics and transporter genotyping could enable personalized baselines [53].

Future neurocritical magnesium assessment must advance beyond total serum limits to multimodal composites: iMg as bioactivity proxy [49], FEMg for renal phenotyping, clinical risks (diuretics, nutrition deficits), and loading tests calibrated to standardized thresholds (tMg ≥ 0.75 mmol/L; iMg ≥ 0.50 mmol/L) against Continuous Renal Replacement Therapy citrate-binding and sepsis losses [11,49].

### 3.4. Magnesium and Outcomes: Evidence Across Neurocritical Syndromes

#### 3.4.1. The ICU-Wide Signal: Dysmagnesemia and Mortality

Dysmagnesemia, encompassing both hypomagnesemia and hypermagnesemia, is common among critically ill patients and consistently emerges as a strong, independent predictor of adverse outcomes. Large ICU and hospitalized cohorts demonstrate a characteristic U-shaped association between serum magnesium and mortality, whereby deviations from the optimal “green zone” (~0.8–1.0 mmol/L total magnesium) correspond with increased risk of death, prolonged ICU stay, and greater need for mechanical ventilation, independent of confounders such as sepsis or renal dysfunction [54,55,56]. Hypomagnesemia, present in over half of ICU admissions, is particularly linked with sepsis, organ failure, and up to a two- to threefold increase in mortality risk [6,54]. In contrast, hypermagnesemia, though less frequent, portends poor prognosis, often reflecting iatrogenic overload or impaired renal clearance, especially in older or multimorbid patients, during ICU stay [57,58]. The emergence of ionized dysmagnesemia during ICU stay may further amplifies mortality risk, underscoring the clinical relevance of dynamic magnesium monitoring [7,59]. These non-linear risks advocate precision-based correction strategies rather than routine, indiscriminate supplementation [60], especially in neurocritical care settings, where disruptions in cerebral homeostasis, due to physiological pathways alterations, can amplify the consequences of electrolyte imbalance.

#### 3.4.2. Acute Ischemic Stroke

Recent literature suggested that hypomagnesaemia can cause more severe ischemic stroke or higher complications related, and it is associated with worse functional outcome at 90 days and one year [20,21,22].

Nonetheless, very few RCTs investigated magnesium’s role as an NMDA antagonist and vasodilator for hyperacute neuroprotection [23,24], and none found a significant reduction in disability or mortality at 90 days, despite differences in dosing strategies and timing of administration. Indeed, the “Intravenous Magnesium Efficacy in Stroke-IMAGES” trial (*n* = 2389;) infused 64 mmol/day MgSO_4_ (started < 12 h onset) vs. placebo, yielding neutral 90-day mRS (modified Rankin Scale). Post hoc subgroup analyses suggested benefit when treatment was initiated within the first six hours or in lacunar strokes, but harm if patients were thrombolyzed, coherent with observational data [23,25,26]. Earlier administration was investigated by the “Field Administration of Stroke Therapy-Magnesium-FAST-MAG” (*n* = 1700; [24]), RCT that pioneered prehospital MgSO_4_ (started < 2 h) in presumed stroke (75% were ischemic), achieving safe ultra-early delivery yet neutral mRS (common OR 1.16, 95% CI 0.95–1.40), even in <1 h subgroup [24]. A recent meta-analyses confirmed neutrality for unselected stroke in functional outcome improvement [27]. These neutral results challenge the concept of magnesium as a universal neuroprotectant but leave open the possibility of benefit in magnesium-deficient patients or with strategies that improve brain magnesium delivery.

#### 3.4.3. Intracerebral Hemorrhage

In the FAST-MAG intracerebral hemorrhage ancillary analyses, administration of prehospital magnesium sulfate showed no reduction in early hematoma expansion or 90-day disability compared with placebo [24]. Magnesium has plausible causal links to reduced hematoma expansion through its physiologic roles as a cofactor in multiple coagulation reactions and in platelet activation and aggregation, supporting thrombin generation and clot stability when serum levels are adequate [28]. In the Naidech ancillary analysis (*n* = 268 with 24 h imaging), initial and follow-up hematoma volumes, hematoma expansion (median 2.0 vs. 1.5 mL), and 3-month modified Rankin Scale distributions were similar between magnesium and placebo, and post-infusion serum magnesium was not correlated with expansion [29]. In a subsequent analysis restricted to patients with both serial imaging and measured magnesium (104 magnesium, 85 placebo, *n* = 189), higher serum magnesium within the magnesium-treated group was associated with lower odds of hematoma expansion and early neurologic deterioration [30]. Moreover, observational data showed an association between admission magnesium levels and both hematoma volume and ICH score [31].

Together with limited prior observational and pilot data, these findings support clinical safety but argue for continued equipoise and for future trials targeting magnesium based on baseline status and achieved serum levels rather than blanket prehospital administration.

#### 3.4.4. Aneurysmal Subarachnoid Hemorrhage

Magnesium has biologically plausible effects in reducing vasospasm in aSAH: it acts as a non-competitive antagonist at voltage-dependent calcium channels and NMDA receptors, promotes cerebrovascular smooth-muscle relaxation via calcium-sensitive potassium channels, and exerts direct vasodilatory effects even in endothelium-impaired, providing a mechanistic basis for vasospasm reduction after aSAH [32,33]. Early phase II studies in aSAH, including MASH-1 [34], suggested that intravenous magnesium could lower angiographic vasospasm and delayed cerebral ischemia (DCI) rates by roughly 20–30%, fueling enthusiasm for large outcome trials.

However, two consecutive phase III trials failed to show benefit: in MASH-2 (*n* = 1204; 64 mmol/day IV MgSO_4_ vs. placebo started < 4 days post-SAH), magnesium did not improve 3-month outcome (mRS ≥ 4 or death; RR 1.03, 95% CI 0.85–1.25), and in IMASH (*n* = 327; 20-mmol loading then 80 mmol/day IV MgSO_4_ titrated to ≈2× baseline up to 2.5 mmol/L for 10–14 days within 48 h of onset), the proportion with favorable 6-month GOS-E was identical between magnesium and placebo (64% vs. 63%; OR 1.0, 95% CI 0.7–1.6), with no differences in mRS, Barthel Index, quality of life, or clinical vasospasm [35,36]. These data were also confirmed by successive metanalysis [36,37].

However, recent observational data indicate that higher serum Mg^2+^ levels (>1.0–1.2 mmol/L) are associated with lower rates of DCI and vasospasm [38,39]. In a 12-year single-center series (*n* = 548), serum-guided magnesium (target 2.0–2.5 mmol/L, days 3–12) yielded DCI rates of 18.8% (vs. 38.3% reduced dose, 51.4% no MgSO_4_; *p* < 0.001) and better long-term mRS (64.7% favorable vs. 50.0%/34.3%; *p* < 0.001) [38]. Moreover, a recent meta-analysis (16 studies) favored MgSO_4_ for angiographic vasospasm (OR 0.48) but confirmed functional neutrality, highlighting potential for exposure-guided strategies [17].

#### 3.4.5. Traumatic Brain Injury

Magnesium has long been considered a plausible neuroprotectant in moderate–severe TBI, but interventional data remain neutral while prognostic signals are increasingly robust. Its pathophysiological rationale is compelling: magnesium blocks NMDA receptor–mediated excitotoxicity, modulates voltage-dependent calcium channels, stabilizes neuronal membranes, and may attenuate blood–brain barrier disruption and cerebral edema, all central components of secondary injury after TBI [40]. In the dedicated magnesium neuroprotection in the TBI trial and the TBI subgroup of the broader IMAGES program (*n* ≈ 1100 combined), 5-day infusions of 64 mmol/day MgSO_4_ initiated within 8 h of injury failed to improve 6-month GOS-E (Glasgow Outcome Scale Extended) (OR 0.84, 95% CI 0.59–1.19; RR for poor outcome 1.00, 95% CI 0.85–1.17), and higher-dose targets were complicated by hypotension and cardiopulmonary adverse events, underscoring the difficulty of dosing in a heterogeneous injured brain [41]. A subsequent Cochrane synthesis of three randomized trials similarly concluded that magnesium confers no clear mortality or disability benefit after acute TBI, and highlighted potential harm at more aggressive dosing, arguing against routine neuroprotective supplementation in unselected patients [42].

In contrast, observational neuro-ICU cohorts repeatedly associate admission magnesium derangements with outcome, associated both to hypo and hypermagnesemia [43,44,45,46]. Large registry analyses show that hypomagnesemia independently predicts higher mortality and worse functional recovery after TBI, with odds of death or GOS-E decline roughly doubled in patients presenting with low magnesium compared with those in the normal range [43,47]. More recent work moves beyond the total serum magnesium toward ionized magnesium as a potentially more patho-physiologically relevant compartment: severe TBI series with continuous multimodal monitoring link low iMg to poorer ICP control, greater secondary insult burden, and worse neurological outcomes, suggesting that ionic rather than total magnesium may better capture the biologically active pool at the neurovascular interface [48,49]. Together, these lines of evidence recast magnesium in TBI less as a proven therapeutic and more as a sensitive prognostic marker, and support a shift toward compartment-specific, status-informed strategies—focusing on iMg and clearly deficient phenotypes—rather than blanket high-dose magnesium for all patients with moderate–severe brain injury.

Overall, RCTs failed to demonstrate statistically significant improvements in their primary clinical outcomes when magnesium was administered to broad, unselected patient populations, compared to placebo. Yet biological rationale, observational gradients, and magnesium signals sustain the hypothesis for precision paradigms targeting verified deficits amid neurocritical vulnerabilities [11,49].

### 3.5. Practical Implications

Neurocritical care clinicians should prioritize magnesium monitoring and targeted repletion in high-risk patients to mitigate complications while avoiding overtreatment, guided by evidence from observational cohorts and trial subgroups [1,10]. This approach aligns with narrative review principles that emphasize a balanced synthesis of evidence, clear clinical translation, and forward-looking recommendations [61]—the major studies considered are summarized in Table 1.

However, ionized magnesium is not universally available, and its implementation is constrained mainly by analyzer availability and non-standardized reference intervals [50,56,62,63].

In settings where ionized magnesium measurement is not available, a pragmatic, tiered approach can be implemented to approximate magnesium status while preserving a precision-oriented framework. First, total serum magnesium should be interpreted systematically in the context of clinical and biochemical modifiers known to affect its reliability, including albumin levels, acid–base status, renal function, and ongoing critical illness–related shifts. Second, dynamic trends rather than single measurements should be prioritized, as intra-patient variability may better reflect biologically relevant changes than absolute values. Third, clinicians may adopt a risk-stratified supplementation strategy, targeting patients at higher risk of intracellular magnesium depletion even in the presence of “normal” total magnesium values. In this context, magnesium administration should be guided by safety thresholds and close monitoring of renal function and hemodynamics. Fourth, the absence of internationally standardized reference intervals for both tMg and iMg represents a fundamental and underappreciated source of measurement uncertainty in neurocritical care. Currently used tMg reference ranges were derived from population distributions rather than clinical outcomes and have been challenged as systematically underestimating the prevalence of deficiency [50,62,63]. Importantly, iMg is not exempt from this limitation: proposed hypomagnesemia cutoffs for serum iMg range from <0.42 to <0.45 mmol/L across published studies [49,50], precluding direct cross-study comparisons. Future research should therefore prioritize the development of outcome-based reference intervals for both tMg and iMg specifically validated in neurocritical care populations, as the adoption of a clinically meaningful threshold may substantially redefine the magnitude and therapeutic relevance of hypomagnesemia in this setting.

Finally, where available, surrogate or adjunctive markers (e.g., urinary magnesium excretion, response to supplementation, or associated electrolyte disturbances such as hypokalemia or hypocalcemia) may help refine clinical judgment. While these approaches do not replace ionized magnesium measurement, they provide a feasible pathway to approximate a precision-based strategy in resource-limited neurocritical care environments.

## 4. Discussion

### 4.1. Who Should Be Monitored More Intensively and How?

Neurocritical patients are often exposed to osmotic therapies (e.g., mannitol), loop diuretics, proton-pump inhibitors, renal replacement therapy, massive transfusions, gastrointestinal losses (e.g., nasogastric suction), or prolonged ICU stays (>7 days), all associated with magnesium loss or shifts. This situation warrants frequent magnesium assessment due to >65% dysmagnesemia (mainly hypomagnesemia) prevalence in these scenarios [1,11]. In these contexts, reliance on existing, highly variable serum tMg reference ranges alone may be insufficient.

Total magnesium measurement may misclassify magnesium status when compared with ionized magnesium (iMg), the biologically active fraction. Across unselected populations, tMg and iMg demonstrate moderate-to-high overall correlation (R^2^ = 0.92, Zwart; R^2^ = 0.70, Yeh; R = 0.64, Soliman et al.) [10,49,59], suggesting reasonable agreement under general conditions. However, this correlation is not uniform across the clinical spectrum and deteriorates substantially in the subgroup where accurate classification is most consequential: in hypomagnesemic ICU patients, Soliman et al. report a markedly attenuated correlation (R = 0.15), indicating that tMg becomes least reliable precisely when a functionally relevant deficit is present [59]. This pattern aligns with prior reports of substantial discordance between tMg- and iMg-based categorical classifications, ranging from approximately 20% to over 60% in critically ill populations [43,47,57]. These primarily reflect differences in categorical assignment (hypo-, normo-, or hypermagnesemia) rather than linear deviation, and are amplified by hypoalbuminemia, acid–base disturbances, and critical illness–related changes in magnesium binding. The clinical implication is not that tMg is globally unreliable, but that its reliability is condition-dependent: it performs adequately in eumagnesemic patients while failing in the exact phenotype targeted by any precision supplementation strategy.

If iMg measure is available, it should be considered in cases of borderline tMg with inconsistent symptoms, high arrhythmia or neuromuscular irritability risk, renal impairment (to prevent hypermagnesemia), or protocolized correction aiming for physiologic targets in research/quality initiatives [11,49]. Point-of-care iMg better predicts outcomes like mortality and ICU length versus tMg [49].

Translating these principles into clinical practice requires a structured, individualized approach rather than empirical supplementation. Hypomagnesemia is the most common magnesium disturbance encountered in the ICU, and when dissociation between total and ionized magnesium occurs, it usually reflects a reduction in the biologically active ionized fraction, meaning that total magnesium may appear within normal range while a functionally relevant deficit goes undetected. As summarized in Figure 1C, management should prioritize the identification of high-risk patients (those receiving diuretics, osmotic therapy, nephrotoxins, or experiencing prolonged gastrointestinal losses), the selection of the appropriate measurement modality and a composite status assessment integrating iMg levels, urinary excretion via FEMg, clinical risk factors, and nutritional magnesium intake.

Where ionized magnesium monitoring is unavailable, clinicians should nonetheless maintain a high index of suspicion, interpret total magnesium with caution, and keep in mind both the limitations of existing RCTs and the plausible neuroprotective mechanisms that make magnesium homeostasis particularly consequential in neurocritical care. Repletion should target verified, clinically meaningful deficits, with targeted correction preferred over blind high-dose supplementation.

### 4.2. Limitations of Current Research

Magnesium sulfate acts both as an essential nutrient substrate and a potential pharmacologic agent. In neurocritical care, however, it has often been approached primarily as the latter while being measured as the former. Major neuroprotection trials in stroke, subarachnoid hemorrhage, and traumatic brain injury overall show that routine magnesium administration does not improve global functional outcomes in unstratified populations, based on serum magnesium levels (Table 1). The reasons for these negative results are likely multifactorial (limited blood–brain barrier penetration, the timing of therapy, dosing strategies, the heterogeneity of patient populations and the absence of personalized therapy) and remain incompletely understood; they should not be attributed solely to measurement bias, even if misclassification of magnesium status is a plausible and clinically relevant contributor.

Conversely, critical care data demonstrate that magnesium disturbances are common, prognostically significant, and frequently misclassified when assessment relies solely on total serum magnesium. This discrepancy highlights a central translational gap between biological relevance and bedside practice.

Three key factors contribute to this gap:

1. Compartment mismatches: The total serum magnesium fails to capture bioactive (ionized) deficiency in 20–85% of ICU patients, leading to frequent misclassification of magnesium status.
2. Patient heterogeneity: Large unselected RCTs using fixed magnesium doses yield negative results, whereas status-informed observational studies reveal graded associations between magnesium levels and outcomes.
3. Pharmacokinetic and pharmacodynamic barriers: Limited blood–brain barrier and blood–CSF barrier penetration may substantially restrict CNS bioavailability of parenterally administered magnesium, with only marginal increases in CSF ionized concentrations reported despite high systemic doses. Beyond bioavailability, the timing of supplementation and inconsistent dosing strategies further reduce the probability of target engagement.
4. Non-standardized reference intervals: Neither tMg nor iMg has an internationally agreed, outcome-based reference range. This definitional inconsistency precludes cross-study comparisons and may systematically misclassify patients as eumagnesemic, independently of which fraction is measured.

Collectively, these factors underscore that patient heterogeneity dilutes trial effects, compartment mismatches confound assessment, and undirected delivery precludes verification of target engagement (e.g., ionized magnesium trajectories, urinary handling, or other biomarkers). Addressing these dimensions may allow magnesium to move from a blunt pharmacologic tool toward a precise metabolic intervention in neurocritical care.

### 4.3. Future Directions

Neurocritical care may gain more by treating magnesium as a status-dependent nutrient–pharmacologic hybrid rather than a universal drug. Future progress hinges on three steps: (1) identifying true bioactive deficiency, (2) quantifying dynamic magnesium debt (losses + shifts − intake), and (3) targeting repletion to biologically relevant compartments and endpoints.

This status-first framework, integrating baseline adequacy, dynamic balance, and ionized magnesium monitoring could offer a coherent way to reconcile conflicting evidence. It reframes magnesium therapy as precision medicine aligned with *Nutrients* priorities: standardized assessment, individual-level evaluation, and linkage to meaningful clinical outcomes.

Future research should prioritize the development of practical bedside tools for precise magnesium status evaluation and mechanistic studies investigating how magnesium sufficiency influences recovery after brain injury. Moving forward, efforts must converge on standardizing magnesium deficiency thresholds and assay reporting, validating ionized magnesium-guided strategies in intensive and neurocritical care settings, and defining composite phenotypes that combine dietary intake, iMg as well as tMg concentrations, and urinary excretion to reflect the full spectrum of magnesium biology.

Additionally, given the established physiological antagonism between magnesium and calcium described in Section 3, and preliminary evidence that serum Mg retains outcome-predictive value in acute ischemic stroke even after adjustment for serum Ca [64], future studies should prospectively evaluate the serum Mg/Ca ratio as a candidate precision biomarker. This ratio may offer a more integrative reflection of functional ionic homeostasis than tMg or iMg alone and could be particularly tractable in resource-limited settings where iMg measurement is unavailable.

Together, these initiatives define a shift toward a status-informed, mechanism-driven paradigm in which magnesium functions as a precision metabolic intervention, transforming biochemical insights into measurable improvements in neurocritical outcomes.

## Figures and Tables

**Table 1:** This table displays key interventional and observational studies on magnesium in neurocritical care: outcomes and signals for precision approaches. Studies prioritized by sample size > 100 or mechanistic insight.

Study (Year) ★ = Key Studies	Design (*n*)	Population	Intervention/Exposure	Primary Outcome	Effect Size (95% CI)	Risk of Bias (Tool)	Overall Signal
**★** Soliman et al. (2003) [59]	Prospective Observational (*n* = 422)	Adult medical–surgical ICU patients	iMg and tMg measured at admission and daily till ICU discharge	Relationship of ionized hypomagnesemia with organ dysfunction, ICU length of stay, and mortality	Mortality OR 1.43 (0.74–2.76)	Moderate (NOS)	iMg hypomagnesemia → worse outcome
**Acute Ischemic Stroke**							
**★** Images (2004) [26]	RCT (*n* = 2389)	Acute ischemic stroke < 12 h	IV MgSO_4_ 64 mmol/day × 5d vs. placebo	90-day mRS shift	OR 0.81 (0.67–0.98) mortality	Low (RoB2)	Neutral (mortality NS after adjustment)
**★** Fast-Mag (2015) [24]	RCT (*n* = 1700)	Presumed stroke < 2 h prehospital (75% ischemic)	IV MgSO_4_ 6 g bolus + 16 g/24 h vs. placebo	90-day mRS shift	cOR 1.16 (0.95–1.40)	Low (RoB2)	Neutral
K. I. Avgerinos et al. (2019) [27]	Meta-analysis (7 RCTs; *n* = 4347)	Acute ischemic stroke	IV MgSO_4_ vs. placebo/standard care	90-day functional outcome (BI, mRS, mortality)	BI > 60: OR 1.05 (0.92–1.19); mRS SMD −0.01 (−0.12 to +0.10); Mortality OR 1.10 (0.94–1.29)	Moderate (GRADE)	Neutral
Q. Xu et al. (2022) [21]	Observational Cohort (*n* = 6483)	AIS with admission hypomagnesemia	Serum Mg level at admission (exposure)	90-day and 1-year mRS	aOR 1.30 (1.02–1.64) for mRS 3–6 at 90d; mortality HR NS after full adjustment	Moderate (NOS)	Hypomagnesemia → worse outcome
**Intracerebral Hemorrhage**							
A.M. Naidech et al. (2022) [29]	RCT ancillary analysis (*n* = 268)	ICH within FAST-MAG; 24 h imaging subgroup	Prehospital MgSO_4_ vs placebo	24 h hematoma expansion (HE)	Difference in HE: 2.0 vs. 1.5 mL (*p* = 0.50)	Some concerns (RoB2; post hoc)	Neutral
**★** E. M. Liotta et al. (2024) [30]	RCT secondary analysis (*n* = 189)	ICH with serial imaging + serum Mg	Serum Mg level (within Mg arm; exposure–response)	HE and neurologic deterioration	aOR 0.64/mg/dL (0.42–0.93)	Moderate (RoB2)	Higher Mg → ↓ HE and ↓ deterioration
**Aneurismal Subarachnoid Hemorrhage**							
MASH-1 (2005) [34]	RCT Phase II (*n* = 283)	Aneurysmal SAH < 4 days	IV MgSO_4_ 80 mmol/day vs. placebo	DCI and poor outcome	DCI: RR 0.66 (95% CI 0.38–1.14); Poor outcome: RR 0.77 (0.54–1.09)—both NS	Some concerns (RoB2; underpowered)	Positive signal → Phase III warranted
**★** MASH-2 (2012) [36]	RCT Phase III (*n* = 1204)	Aneurysmal SAH < 4 days	IV MgSO_4_ 64 mmol/day × 14d vs. placebo	3-month poor outcome (mRS ≥ 4 or death)	RR 1.03 (0.85–1.25)	Low (RoB2)	Neutral
C. Wipplinger et al. (2023) [38]	Retrospective cohort (*n* = 548)	aSAH; serum-guided Mg supplementation (3 groups)	Target serum Mg 2.0–2.5 mmol/L (days 3–12) vs. lower/no Mg	DCI rate and long-term mRS	DCI: 18.8% vs. 38.3% vs. 51.4% (*p* < 0.001) mRS fav: 64.7% vs. 50.0% vs. 34.3% (*p* < 0.001)	Moderate (NOS; single center)	Target Mg → ↓ DCI ↑ favorable outcome
**★** Zheng et al. (2023) [17]	Meta-analysis (16 studies; *n* = 3503)	Aneurysmal SAH	IV MgSO_4_ vs. placebo/control	Vasospasm and functional outcome	Vasospasm: OR 0.48 Functional: neutral	Moderate (GRADE)	↓ Vasospasm; functional outcome neutral
IMASH (2010) [35]	RCT Phase III (*n* = 327)	Aneurysmal SAH < 48 h; 10 multicenter sites (Asia-Pacific)	IV MgSO_4_ 20 mmol bolus + 80 mmol/day titrated to 2× baseline serum Mg ×10–14d vs. saline placebo	6-month favorable outcome (GOSE 5–8)	OR 1.0 (0.7–1.6)	Low (RoB2)	Neutral; no benefit in any subgroup
D. Reddy et al. (2014) [37]	Meta-analysis (10 RCTs; *n* = 2174 for functional outcomes)	Aneurysmal SAH	IV MgSO_4_ vs. placebo (variable dose/duration across studies)	Functional outcome (GOS/MRS), DCI, mortality	Functional (GOS/MRS): RR 0.93 (0.82–1.06); Mortality: RR 0.95 (0.76–1.17); DCI: RR 0.54 (0.38–0.75)	High (GRADE)	↓ DCI (significant, moderate-quality evidence); functional outcome and mortality neutral (high-quality evidence)
**Traumatic Brain Injury**							
**★** N. R. Temkin et al. (2007) [41]	RCT (*n* = 499)	Moderate/severe TBI < 8 h; two Mg target ranges	IV MgSO_4_ 64 mmol/day × 5d vs. placebo	6-month GOS-E favorable outcome	RR poor outcome 1.00 (0.85–1.17)	Low (RoB2)	Neutral; ↑ adverse events at higher doses
A.M. Bainbridge D et al. (2022) [42]	Cochrane Meta-analysis (3 RCTs; *n* = 574)	Moderate/severe TBI	IV MgSO_4_ vs. placebo	Mortality and disability	Mortality RR 1.48 (1.00–2.19), *p* = 0.05 (1 study); GOS MD 0.02 (−0.38 to +0.41), *p* = 0.94 (3 studies)	Moderate (GRADE)	No benefit; possible harm at high doses
M. Stippler et al. (2007) [46]	Cohort (*n* = 216)	Severe TBI with CSF Mg measurement	Serum and CSF Mg (exposure; observational)	Long-term prognosis (GOS)	OR 2.37 (1.18–4.78) low serum Mg; OR 7.63 high CSF Mg	Moderate (NOS)	Low admission Mg → worse outcome
**★** R. Wang et al. (2022) [43]	Registry cohort (*n* = 2280)	Severe TBI (multicenter registry)	Admission hypomagnesemia vs. normo-magnesemia	Mortality and GOS-E	U-shaped: both hypo- and hypermagnesemia → ↑ mortality; aOR NR	Moderate (NOS; registry data)	U-shaped Mg–mortality relationship; extreme values (both low and high) → ↑ risk
E. Jung et al. (2025) [47]	Cohort (*n* = 482)	TBI (mixed severity); admission Mg	Serum Mg at admission (exposure)	Long-term mortality	OR 1.80 (1.14–2.68) disability; OR 1.82 (1.03–2.99) mortality	Moderate (NOS)	Low Mg → independently ↑ mortality
S. Bal et al. (2024) [48]	Cohort (*n* = 84)	Severe TBI; total Mg (tMg) monitoring	tMg level vs. ICP control and outcome	Neurological outcome and ICP control	Low admission Mg (<1.6 mEq/L): OR 6.59, *p* = 0.002 for poor 6-month GOS	Moderate (NOS; small N single-center)	Low iMg < 0.5 mmol/L → worse ICP control and outcome
**Preclinical**							
**★** F. C. P. Ribeiro et al. (2025) [40]	Meta-analysis of animal studies (16 studies)	Rat TBI (diffuse axonal injury and contusion)	NMDA antagonists (including MgSO_4_)	Brain edema, behavioral recovery, iMg restoration	Consistent neuroprotection (not poolable)	Moderate (SYRCLE/CAMARADES)	Early Mg linked to ↓ edema, ↑ recovery; iMg restoration correlates with benefit; timing critical

★ Key study: selected based on sample size ≥ 500, Phase III RCT design, meta-analysis, or high analytical impact on the field. Newcastle-Ottawa Scale (NOS) for observational studies; GRADE for meta-analyses/systematic reviews in humans and SYRCLE/CAMARADES in mice. Assessments are approximate and should be verified with formal primary appraisal. Effect sizes: OR = odds ratio; RR = relative risk; aOR = adjusted OR; cOR = common OR; 95% CI = 95% confidence interval; NR = not reported. Abbreviations: AIS = acute ischemic stroke; aSAH = aneurysmal subarachnoid hemorrhage; DCI = delayed cerebral ischemia; BI = Barthel Index; GOS = Glasgow Outcome Scale; GOS-E = Glasgow Outcome Scale Extended (favorable ≥ 5); HE = hematoma expansion; ICH = intracerebral hemorrhage; iMg = ionized magnesium; ICP = intracranial pressure; mRS = modified Rankin Scale (0–2 favorable); SAH = subarachnoid hemorrhage; TBI = traumatic brain injury; CSF = CerebroSpinal Fluid. Directional symbols: ↑ = increase or improvement; ↓ = decrease or reduction; → = leads to/associated with.

## Data Availability

No new data were created or analyzed in this study. Data sharing is not applicable to this article.

## Abbreviations

The following abbreviations are used in this manuscript:

SAH	Subarachnoid Aneurysmal Hemorrhage
TBI	Traumatic Brain Injury
ICH	Intra Cerebral Hemorrhage
iMg	Ionized Magnesium
tMg	Total Magnesium
MgSO_4_	Magnesium Sulfate
DCI	Delayed Cerebral Ischemia
FEMg	Fractional excretion of magnesium
GOS-E	Glasgow Outcome Scale Extended
ICU	Intensive care unit
mRS	modified Rankin Scale
NMDA	N-methyl-D-aspartate
OR	Odds ratio
RCT	Randomized controlled trial
RR	Relative risk
SANRA	Scale for the Assessment of Narrative Review Articles

## Appendix A

(“Magnesium”[MeSH Terms] OR “magnesium”[tiab] OR “ionized magnesium”[tiab] OR “Magnesium Sulfate”[MeSH Terms]) AND (“Brain Injuries, Traumatic”[MeSH Terms] OR “traumatic brain injur*”[tiab] OR “TBI”[tiab] OR “Subarachnoid Hemorrhage”[MeSH Terms] OR “subarachnoid hemorrhage”[tiab] OR “subarachnoid haemorrhage”[tiab] OR “SAH”[tiab] OR “Cerebral Hemorrhage”[MeSH Terms] OR “intracerebral hemorrhage”[tiab] OR “intracerebral haemorrhage”[tiab] OR “ICH”[tiab] OR “Ischemic Stroke”[MeSH Terms] OR “Brain Ischemia”[MeSH Terms] OR “ischemic stroke”[tiab] OR “ischaemic stroke”[tiab] OR “Vasospasm, Intracranial”[MeSH Terms] OR “vasospasm”[tiab] OR “delayed cerebral ischemia”[tiab] OR “delayed cerebral ischaemia”[tiab] OR “DCI”[tiab] OR “neurocritical care”[tiab] OR “neurointensive care”[tiab]) AND (“1984/01/01”[PDAT]: “2025/12/31”[PDAT])

## References

[1] Saglietti F., Girombelli A., Marelli S., Vetrone F., Balzanelli M.G., Tabaee Damavandi P. (2023). Role of Magnesium in the Intensive Care Unit and Immunomodulation: A Literature Review. Vaccines.

[2] Polderman K.H., Bloemers F.W., Peerdeman S.M., Girbes A.R.J. (2000). Hypomagnesemia and hypophosphatemia at admission in patients with severe head injury. Crit. Care Med..

[3] Salinas M., López-Garrigós M., Flores E., Leiva-Salinas C. (2024). Improving diagnosis and treatment of hypomagnesemia. Clin. Chem. Lab. Med..

[4] Patel V., Akimbekov N.S., Grant W.B., Dean C., Fang X., Razzaque M.S. (2024). Neuroprotective effects of magnesium: Implications for neuroinflammation and cognitive decline. Front. Endocrinol..

[5] Hansen B.-A., Bruserud Ø. (2018). Hypomagnesemia in critically ill patients. J. Intensive Care.

[6] Upala S., Jaruvongvanich V., Wijarnpreecha K., Sanguankeo A. (2016). Hypomagnesemia and mortality in patients admitted to intensive care unit: A systematic review and meta-analysis. Int. J. Med..

[7] Escuela M.P., Guerra M., Añón J.M., Martínez-Vizcaíno V., Zapatero M.D., García-Jalón A., Celaya S. (2005). Total and ionized serum magnesium in critically ill patients. Intensive Care Med..

[8] Huijgen H.J., Soesan M., Sanders R., Mairuhu W.M., Kesecioglu J., Sanders G.T. (2000). Magnesium Levels in Critically Ill Patients. Am. J. Clin. Pathol..

[9] Johansson M., Whiss P.A. (2007). Weak Relationship Between Ionized and Total Magnesium in Serum of Patients Requiring Magnesium Status. BTER.

[10] Yeh D.D., Chokengarmwong N., Chang Y., Yu L., Arsenault C., Rudolf J., Lee-Lewandrowski E., Lewandrowski K. (2017). Total and ionized magnesium testing in the surgical intensive care unit—Opportunities for improved laboratory and pharmacy utilization. J. Crit. Care.

[11] Touyz R.M., De Baaij J.H.F., Hoenderop J.G.J. (2024). Magnesium Disorders. N. Engl. J. Med..

[12] Mayer M.L., Westbrook G.L., Guthrie P.B. (1984). Voltage-dependent block by Mg^2+^ of NMDA responses in spinal cord neurones. Nature.

[13] Nowak L., Bregestovski P., Ascher P., Herbet A., Prochiantz A. (1984). Magnesium gates glutamate-activated channels in mouse central neurones. Nature.

[14] Huang X., Sun X., Wang Q., Zhang J., Wen H., Chen W.-J., Zhu S. (2025). Structural insights into the diverse actions of magnesium on NMDA receptors. Neuron.

[15] Cox J.A., Lysko P.G., Henneberry R.C. (1989). Excitatory amino acid neurotoxicity at the N-methyl-D-aspartate receptor in cultured neurons: Role of the voltage-dependent magnesium block. Brain Res..

[16] Lipton P. (1999). Ischemic cell death in brain neurons. Physiol. Rev..

[17] Zheng H., Guo X., Huang X., Xiong Y., Gao W., Ke C., Chen C., Pan Z., Ye L., Wang L. (2023). Effect of magnesium sulfate on cerebral vasospasm in the treatment of aneurysmal subarachnoid hemorrhage: A systematic review and meta-analysis. Front. Neurol..

[18] Kudryavtseva O., Lyngsø K.S., Jensen B.L., Dimke H. (2024). Nitric oxide, endothelium-derived hyperpolarizing factor, and smooth muscle-dependent mechanisms contribute to magnesium-dependent vascular relaxation in mouse arteries. Acta Physiol..

[19] Murata T., Dietrich H.H., Horiuchi T., Hongo K., Dacey R.G. (2016). Mechanisms of magnesium-induced vasodilation in cerebral penetrating arterioles. Neurosci. Res..

[20] Xu R., Wang L., Sun L., Dong J. (2021). Neuroprotective effect of magnesium supplementation on cerebral ischemic diseases. Life Sci..

[21] Xu Q., Hu L., Chen L., Li H., Tian X., Zuo Y., Zhang Y., Zhang X., Sun P., Wang Y. (2023). Low serum magnesium is associated with poor functional outcome in acute ischemic stroke or transient ischemic attack patients. CNS Neurosci. Ther..

[22] Shadman J., Sadeghian N., Moradi A., Bohlooli S., Panahpour H. (2019). Magnesium sulfate protects blood–brain barrier integrity and reduces brain edema after acute ischemic stroke in rats. Metab. Brain Dis..

[23] Intravenous Magnesium Efficacy in Stroke (IMAGES) Study Investigators (2004). Magnesium for acute stroke (Intravenous Magnesium Efficacy in Stroke trial): Randomised controlled trial. Lancet.

[24] Saver J.L., Starkman S., Eckstein M., Stratton S.J., Pratt F.D., Hamilton S., Conwit R., Liebeskind D.S., Sung G., Kramer I. (2015). Prehospital Use of Magnesium Sulfate as Neuroprotection in Acute Stroke. N. Engl. J. Med..

[25] Qiu H., Shen R., Chen L., Pandey S., Sun J., Deng H. (2022). Low Serum Magnesium Levels Are Associated with Hemorrhagic Transformation After Mechanical Thrombectomy in Patients with Acute Ischemic Stroke. Front. Neurol..

[26] Aslanyan S., Weir C.J., Muir K.W., Lees K.R. (2007). Magnesium for Treatment of Acute Lacunar Stroke Syndromes: Further Analysis of the IMAGES Trial. Stroke.

[27] Avgerinos K.I., Chatzisotiriou A., Haidich A.-B., Tsapas A., Lioutas V.-A. (2019). Intravenous Magnesium Sulfate in Acute Stroke: A Systematic Review and Meta-Analysis of Randomized Controlled Trials. Stroke.

[28] Chang J., Armonda R., Goyal N., Arthur A. (2019). Magnesium: Pathophysiological mechanisms and potential therapeutic roles in intracerebral hemorrhage. Neural Regen. Res..

[29] Naidech A.M., Shkirkova K., Villablanca J.P., Sanossian N., Liebeskind D.S., Sharma L., Eckstein M., Stratton S., Conwit R., Hamilton S. (2022). Magnesium Sulfate and Hematoma Expansion: An Ancillary Analysis of the FAST-MAG Randomized Trial. Stroke.

[30] Liotta E.M., Maas M.B., Prabhakaran S., Shkirkova K., Sanossian N., Liebeskind D.S., Sharma L., Stratton S., Conwit R., Saver J.L. (2024). Magnesium and Hematoma Expansion in Intracerebral Hemorrhage: A FAST-MAG Randomized Trial Analysis. Stroke.

[31] Goyal N., Tsivgoulis G., Malhotra K., Houck A.L., Khorchid Y.M., Pandhi A., Inoa V., Alsherbini K., Alexandrov A.V., Arthur A.S. (2018). Serum Magnesium Levels and Outcomes in Patients with Acute Spontaneous Intracerebral Hemorrhage. JAHA.

[32] Sommer B., Weidinger C.S., Wolf D., Buchfelder M., Schmitt H. (2017). Intraoperative continuous cerebral microcirculation measurement in patients with aneurysmal subarachnoid hemorrhage: Preliminary data on the early administration of magnesium sulfate. BMC Anesth..

[33] Nishida S., Kawauchi S., Toyooka T., Kumagai K., Takeuchi S., Sato S., Kondo A., Wada K., Mori K. (2021). Local Application of Magnesium Sulfate Solution Suppressed Cortical Spreading Ischemia and Reduced Brain Damage in a Rat Subarachnoid Hemorrhage-Mimicking Model. World Neurosurg..

[34] Van Den Bergh W.M. (2005). Magnesium Sulfate in Aneurysmal Subarachnoid Hemorrhage: A Randomized Controlled Trial. Stroke.

[35] Wong G.K.C., Poon W.S., Chan M.T.V., Boet R., Gin T., Ng S.C.P., Zee B.C.Y. (2010). Intravenous Magnesium Sulphate for Aneurysmal Subarachnoid Hemorrhage (IMASH): A Randomized, Double-Blinded, Placebo-Controlled, Multicenter Phase III Trial. Stroke.

[36] Mees S.M.D., Algra A., Vandertop W.P., Van Kooten F., Kuijsten H.A., Boiten J., Van Oostenbrugge R.J., Salman R.A.-S., Lavados P.M., Rinkel G.J. (2012). Magnesium for aneurysmal subarachnoid haemorrhage (MASH-2): A randomised placebo-controlled trial. Lancet.

[37] Reddy D., Fallah A., Petropoulos J.-A., Farrokhyar F., Macdonald R.L., Jichici D. (2014). Prophylactic Magnesium Sulfate for Aneurysmal Subarachnoid Hemorrhage: A Systematic Review and Meta-analysis. Neurocrit. Care.

[38] Wipplinger C., Cattaneo A., Wipplinger T.M., Lamllari K., Semmler F., Geske C., Messinger J., Nickl V., Beez A., Ernestus R.-I. (2023). Serum concentration–guided intravenous magnesium sulfate administration for neuroprotection in patients with aneurysmal subarachnoid hemorrhage: A retrospective evaluation of a 12-year single-center experience. Neurosurg. Rev..

[39] Feulner J., Weidinger C.S., Dörfler A., Birkholz T., Buchfelder M., Sommer B. (2024). Early Intravenous Magnesium Sulfate and Its Impact on Cerebral Vasospasm as well as Delayed Cerebral Ischemia in Aneurysmal Subarachnoid Hemorrhage: A Retrospective Matched Case-Control Analysis. World Neurosurg..

[40] Ribeiro F.C.P., De Oliveira N.V., Coral G.R., De Assis César A.R., Gonçalves M.W.A., Egal E.S.A., Pereira K.F. (2025). Efficacy of N-Methyl-D-Aspartate (NMDA) Receptor Antagonists in Treating Traumatic Brain Injury–Induced Brain Edema: A Systematic Review and Meta-analysis of Animal Studies. Neurocrit. Care.

[41] Temkin N.R., Anderson G.D., Winn H.R., Ellenbogen R.G., Britz G.W., Schuster J., Lucas T., Newell D.W., Mansfield P.N., Machamer J.E. (2007). Magnesium sulfate for neuroprotection after traumatic brain injury: A randomised controlled trial. Lancet Neurol..

[42] Arango M.F., Bainbridge D. (2008). Magnesium for acute traumatic brain injury. Cochrane Database Syst. Rev..

[43] Wang R., He M., Xu J. (2022). Initial Serum Magnesium Level Is Associated with Mortality Risk in Traumatic Brain Injury Patients. Nutrients.

[44] Nayak R., Attry S., Ghosh S. (2018). Serum magnesium as a marker of neurological outcome in severe traumatic brain injury patients. Asian J. Neurosurg..

[45] Mekkodathil A., El-Menyar A., Hakim S., Al Jogol H., Parchani A., Peralta R., Rizoli S., Al-Thani H. (2023). Initial Serum Levels of Magnesium and Calcium as Predictors of Mortality in Traumatic Brain Injury Patients: A Retrospective Study. Diagnostics.

[46] Stippler M., Fischer M.R., Puccio A.M., Wisniewski S.R., Carson-Walter E.B., Dixon C.E., Walter K.A. (2007). Serum and Cerebrospinal Fluid Magnesium in Severe Traumatic Brain Injury Outcome. J. Neurotrauma.

[47] Jung E., Lee J.-H., Park H.-K. (2025). Association between serum magnesium level and long-term prognosis of traumatic brain injury. Brain Inj..

[48] Bal S., Jain S., Acharya S., Gupta A. (2025). Effect of Serum Ionic Magnesium on Neurological Outcome in Severe Traumatic Brain Injury Patients: A Prospective Study. Indian J. Neurotrauma.

[49] Zwart J.P., Zwartkruis M., Van Borren M.M.G.J., Van Vliet J., Bech A.P. (2025). Paradigm shift in hypomagnesemia: A prospective observational study of ionized magnesium in the ICU. Crit. Care.

[50] Costello R.B., Elin R.J., Rosanoff A., Wallace T.C., Guerrero-Romero F., Hruby A., Lutsey P.L., Nielsen F.H., Rodriguez-Moran M., Song Y. (2016). Perspective: The Case for an Evidence-Based Reference Interval for Serum Magnesium: The Time Has Come. Adv. Nutr..

[51] Tong G.M., Rude R.K. (2005). Magnesium Deficiency in Critical Illness. J. Intensive Care Med..

[52] Rude R.K. (1998). Magnesium Deficiency: A Cause of Heterogenous Disease in Humans. J. Bone Miner. Res..

[53] De Baaij J.H.F., Hoenderop J.G.J., Bindels R.J.M. (2015). Magnesium in Man: Implications for Health and Disease. Physiol. Rev..

[54] Jiang P., Lv Q., Lai T., Xu F. (2017). Does Hypomagnesemia Impact on the Outcome of Patients Admitted to the Intensive Care Unit? A Systematic Review and Meta-Analysis. Shock.

[55] Fairley J., Glassford N.J., Zhang L., Bellomo R. (2015). Magnesium status and magnesium therapy in critically ill patients: A systematic review. J. Crit. Care.

[56] Malinowska J., Małecka M., Ciepiela O. (2020). Variations in Magnesium Concentration Are Associated with Increased Mortality: Study in an Unselected Population of Hospitalized Patients. Nutrients.

[57] Cheungpasitporn W., Thongprayoon C., Qian Q. (2015). Dysmagnesemia in Hospitalized Patients: Prevalence and Prognostic Importance. Mayo Clin. Proc..

[58] Akin Şen İ. (2025). Prognostic value of admission serum magnesium and systemic inflammation indices in septic ICU patients aged 85 years and older: A retrospective observational study. Medicine.

[59] Soliman H.M., Mercan D., Lobo S.S.M., Mélot C., Vincent J.-L. (2003). Development of ionized hypomagnesemia is associated with higher mortality rates. Crit. Care Med..

[60] Goulden R., Abrahamowicz M., Strumpf E., Tamblyn R. (2026). Magnesium Supplementation and Tachyarrhythmias: A Nonrandomized Clinical Trial. JAMA Intern. Med..

[61] Baethge C., Goldbeck-Wood S., Mertens S. (2019). SANRA—A scale for the quality assessment of narrative review articles. Res. Integr. Peer Rev..

[62] Liebscher D.H., Liebscher D.E. (2004). About the misdiagnosis of magnesium deficiency. J. Am. Coll. Nutr..

[63] Rosanoff A., West C., Elin R.J., Micke O., Baniasadi S., Barbagallo M., Campbell E., Cheng F.-C., Costello R.B., Gamboa-Gomez C. (2022). Recommendation on an updated standardization of serum magnesium reference ranges. Eur. J. Nutr..

[64] Feng P., Niu X., Hu J., Zhou M., Liang H., Zhang Y., Tong W., Xu T. (2013). Relationship of serum magnesium concentration to risk of short-term outcome of acute ischemic stroke. Blood Press..

